# Uncovering the role of chemical pollutants in shaping biological invasions

**DOI:** 10.1098/rspb.2025.1232

**Published:** 2025-10-22

**Authors:** Isaac Y. Ligocki, Jack A. Brand, Eli S. J. Thoré, Upama Aich, Tomas Brodin, Morelia Camacho-Cervantes, Jake M. Martin, Amelia Munson, Giovanni Polverino, Bob B. M. Wong, Michael G. Bertram

**Affiliations:** ^1^ Department of Zoology and Physiology, University of Wyoming, Laramie, WY, USA; ^2^ Department of Biology, Millersville University of Pennsylvania, Millersville, PA, USA; ^3^ Department of Wildlife, Fish, and Environmental Studies, Swedish University of Agricultural Sciences, Umeå, Sweden; ^4^ Institute of Zoology, Zoological Society of London, London, UK; ^5^ Laboratory of Adaptive Biodynamics, Research Unit of Environmental and Evolutionary Biology, Institute of Life, Earth, and Environment, University of Namur, Namur, Belgium; ^6^ TRANSfarm Science, Engineering, and Technology Group, Lovenjoel, Leuven, Belgium; ^7^ School of Biological Sciences, Monash University, Melbourne, Victoria, Australia; ^8^ School for Biological Sciences, Centre for Evolutionary Biology, The University of Western Australia, Perth, Western Australia, Australia; ^9^ Instituto de Ciencias del Mar y Limnología, Universidad Nacional Autónoma de México, Mexico City, Mexico; ^10^ School of Life and Environmental Sciences, Deakin University, Geelong, Victoria, Australia; ^11^ School of Agriculture, Biomedicine and Environment, La Trobe University, Melbourne, Victoria, Australia; ^12^ Department of Ecological and Biological Sciences, University of Tuscia, Viterbo, Italy; ^13^ Department of Zoology, Stockholm University, Stockholm, Sweden

**Keywords:** contaminant, direct and indirect effects, ecological impact, ecotoxicology, invasive species, toxicity

## Abstract

Ecosystems around the globe are under unprecedented pressure from human activities. Chemical pollution and biological invasions are two leading drivers of environmental change, each of which causes substantial harm to wildlife and the ecosystems they inhabit. However, despite their individual impacts being well-documented, the combined effects of these pervasive environmental pressures are seldom studied. Here, we address this critical gap by first examining the potential interactions between chemical pollution and biological invasions in animals. We then discuss possible impacts of chemical pollution on animals—both invasive and native—across the distinct stages of the invasion process. Further, we examine gaps in our current understanding of the potential interactions between chemical pollution and biological invasions, including the role of pollutants in mediating interactions between native and non-native species, how pollutants may influence the potential for the invasion process to act as a selective filter, and the relevance of phenotypic plasticity and behavioural syndromes in this context. By synthesizing current knowledge and identifying key research gaps, this review underscores the importance of considering chemical pollution and biological invasions in combination in ecological research. Understanding the combined impacts of these widespread and frequently co-occurring phenomena is essential for developing effective conservation and restoration measures in an increasingly human-modified world.

## 1. Introduction

The introduction, establishment and spread of invasive species is one of the most pressing challenges for wildlife globally [1]. These invasions have been reported to cost the global economy US$26.8 billion per annum in management and mitigation, with the cost only expected to grow over the coming decades [2]. Invasive species may impact native wildlife as competitors [3], predators [4], vectors of pathogens [5] or even toxic items that are perceived as prey [6]. In this regard, we define invasive species as organisms that are deliberately or accidentally introduced outside of their historical range, have established a self-sustaining population, and are actively spreading through the landscape [7]. While there is no shortage of documented cases of the catastrophic impacts of invasive species on native wildlife (e.g. [8]), communities are often dynamic in their response to biological invasions. The impact of the introduction of a non-native species may range from disrupted predator–prey relationships to more complex changes within an ecosystem, such as shifts in community structure [8,9] or changes in nutrient cycling [10,11].

There is growing evidence that the successful establishment and expansion of invasive species, as well as native wildlife’s resilience to them, can be influenced by the traits of the invaders. Different phenotypes can facilitate progression through the successive stages of species invasion, from departure from the current patch to movement between patches (transience) and settlement in a new patch. A growing body of research has identified patterns of shared phenotypic traits associated with successful invasive individuals and species [12–14]. While not homogeneous across taxa, some frequently described traits of successful invaders include increased limb size [15], greater thermal physiological tolerance [16], and life-history traits such as fast development to sexual maturity and high fertility [17,18], as well as behavioural traits such as high activity and dispersal propensity [19], low sociality [20] and increased boldness [21].

While rarely studied in the context of invasion ecology, many of the morphological, life-history and behavioural traits that influence biological invasions are known to be moderated by physiological processes that are vulnerable to chemical pollutants [22,23]. In this review, we define chemical pollutants as synthetic (e.g. pesticides, herbicides, fungicides, per- and polyfluoroalkyl substances (PFAS), plasticizers, polychlorinated biphenyls (PCBs), dioxins, pharmaceuticals and personal care products) or naturally occurring compounds (e.g. heavy metals, nitrates and nitrites, phosphates) that have been introduced or mobilized into environments as a result of human activities, and which can negatively impact organisms and ecosystems by interfering with biological processes. Chemical pollutants are widespread globally and have been found even in the most isolated regions of the planet [24]. Indeed, thousands of chemical pollutants, including those present in industrial waste, pesticides and pharmaceuticals have been detected in water, soil and air, as well as in wildlife [25,26] and humans [27].

Studies directly investigating the interactions between chemical pollution and invasions by animals are few and far between (but see [28,29]). This contrasts with the considerable research attention that has been paid to the potential for herbicide use to influence the spread of invasive plants via native plant suppression [30,31], as well as the interactive effects of other human-induced environmental impacts and invasion [32,33]. Nevertheless, despite the relatively few animal studies examining the interaction between biological invasions and chemical pollution, we can forecast the resulting outcomes based on a growing body of literature reporting pollutant-induced impacts on a wide range of key phenotypic traits [34,35]. Indeed, multiple independent classes of pollutants have been reported to moderate behavioural traits such as activity/exploration [36,37], boldness [38,39], sociability [36,40] and/or aggressiveness [41,42]. Similarly, many pollutants can alter metabolism [43], growth [44], fecundity [44,45] and body condition [46]. While the direction and magnitude of these effects, and their mechanism(s) of action, can vary greatly from pollutant to pollutant, it is clear that exposure to chemical pollutants can mediate the expression of the same phenotypic traits that contribute to successful invasions.

In this review, we explore the potential for interactions between exposure to chemical pollutants and invasion of non-native animal species. To date, only a few studies have empirically investigated the relationship between these two pervasive forms of human-induced environmental change. As such, we explore and highlight mechanisms through which chemical pollutants have been shown to influence the biology of animals in ways that are expected to moderate the success of invasive populations at the (i) transport, (ii) introduction, (iii) establishment and (iv) spread stages of the invasion process [47]. Building upon this foundation, we then explore emerging areas of research that we anticipate will further illuminate the potential mechanisms and processes through which chemical pollutants and the invasion of non-native species may interact in nature.

## 2. Chemical pollution impacts throughout the invasion process

**Figure 1.**
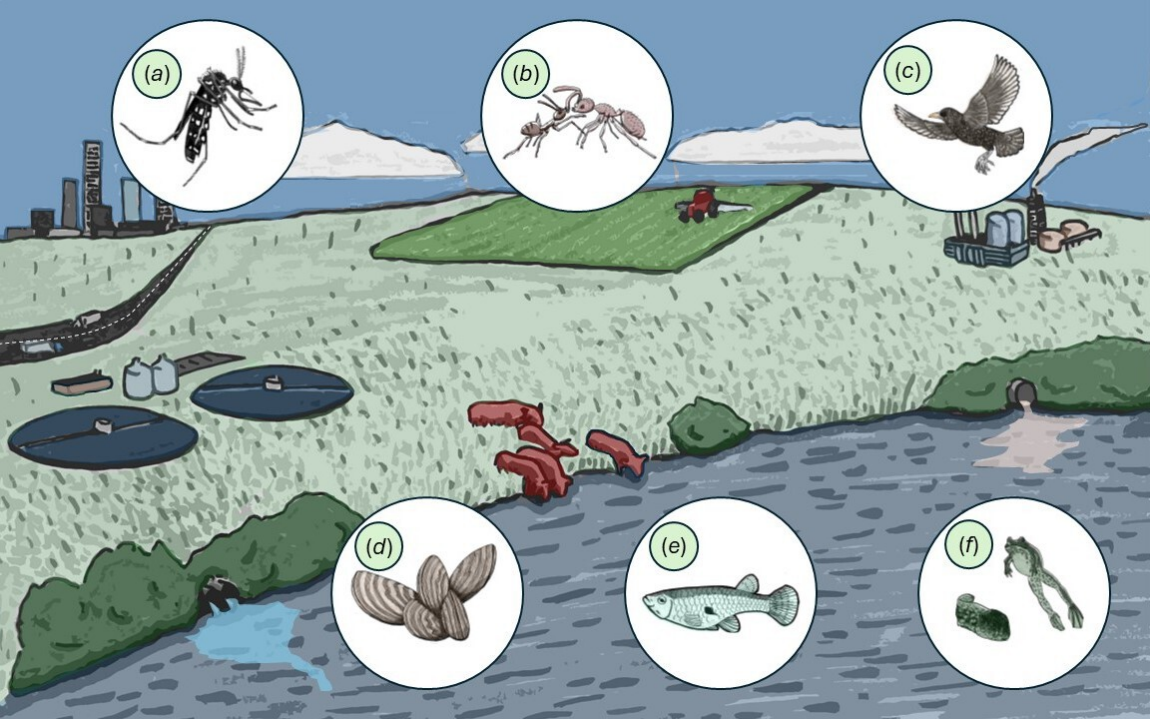
Notable examples of pollutant-moderated effects on phenotypes associated with biological invasions in widely introduced species. (*a*) Contamination from tyre particle leachates facilitated the persistence of a native common house mosquito (*Culex pipiens*) in the presence of an invasive Asian tiger mosquito (*Aedes albopictus*) [50]. (*b*) A neurotoxic pesticide increased the aggression of Argentine ants (*Linepithema humile*) towards unexposed native southern ants (*Monomorium antarcticum*) [51]. (*c*) European starlings (*Sturnus vulgaris*)—a widespread invader—exposed to polychlorinated biphenyls (PCBs) were slower and more error-prone at spatial learning tasks [52]. (*d*) A mixture of illicit drugs at realistic environmental concentrations impaired the oxidative status of zebra mussels (*Dreissena polymorpha*) [53]. (*e*) Female eastern mosquitofish (*Gambusia holbrooki*) exposed to the endocrine-disrupting chemical 17β-trenbolone were more active and exploratory in a novel environment [54]. (*f*) Perfluorooctane sulfonate (PFOS) and perfluorooctanoic acid (PFOA) altered mass and delayed development in invasive American bullfrogs (*Lithobates catesbeiana*) [55].

**Figure 2.**
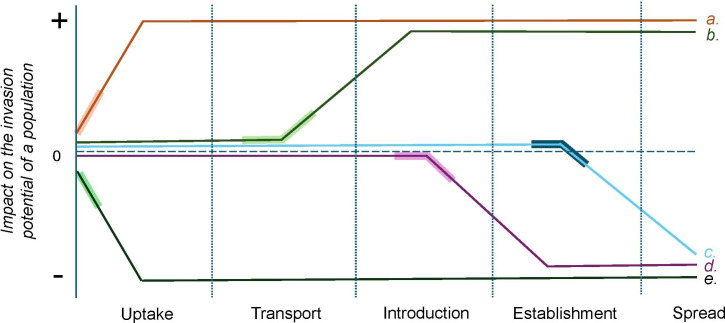
The hypothetical impact of chemical pollution exposure on the invasion potential of a population. Bold regions of each path indicate the stage in which exposure takes place. (*a*) Exposure of a population within its native range to a chemical pollutant that increases reproduction may increase the likelihood of uptake into a transport vector. (*b*) A population is exposed during transport to a chemical that increases their thermal tolerance and likelihood of surviving this stage. (*c*) An established population is unable to expand their range beyond the initial introduction site because neighbouring habitats are polluted, preventing spread and subsequent colonization. (*d*) A group of organisms is successfully introduced into a novel habitat that contains a pollutant that reduces growth rate and they are unable to outcompete native organisms that have already adapted to the pollutant in order to establish a viable population. (*e*) A pollutant reduces the activity levels of organisms in their native range, and as a result uptake into a transport vector is less likely. Note that this graphic conveys hypothetical scenarios where populations are exposed only once during the invasion process, and where the effects of pollutant exposure are only either positive or negative for invasion potential. In nature, interactions between chemical pollution and biological invasions are likely to be more complex, with positive, neutral or negative effects of exposure potentially occurring across multiple stages of invasion.

The invasion process is made up of multiple sequential stages (uptake, transport, introduction, establishment and spread) during which an invading species can either succeed or fail [47,48]. Much research has demonstrated that various phenotypic traits play a key role in mediating the ability of organisms to progress through the invasion process [7,48,49]. Importantly, chemical pollutants have been shown to impact many of the same traits that are associated with invasion success within and across these stages (figure 1). Below, we discuss how pollutants may impact the success of species across each of the stages of invasion. These pollutant-driven impacts on organisms and populations may have positive or negative impacts on the success of particular stages of the invasion process (figure 2).

### (a) Uptake, transport and introduction

Prior to establishment in a new area, animals must first be transported and introduced. This process is often broken up into three stages—uptake, transport and introduction—each of which can select for a subset of individuals with non-random life-history, morphological, physiological and behavioural traits [48,49,56]. Historically, the initial stages of invasion have been less of a focus of research compared with understanding the traits that promote establishment or spread in a new area. However, recent invasion frameworks have emphasized these stages as particularly important because they act as the first filter in the invasion process at which individuals can succeed or fail [48,57], and can limit the amount of trait variation available at later stages. These early stages of the invasion process may be more likely to occur in areas of intense human activity, which are areas that also may experience a higher load of chemical pollutants [58].

Propagule pressure—the total number of individuals entering a new area—is a key determinant of invasion success, with much research reporting a positive relationship between introduction success and the number of individuals that are taken up into transport vectors and released into non-native environments [59]. Thus, any chemical pollutant that causes widespread mortality and the collapse of native populations may drastically reduce the opportunity for those species to enter transport pathways and become invasive. Many pollutants can lead to mortality, including chemicals that are specifically designed to kill animals (e.g. insecticides [60]) but also chemicals that were not designed for this purpose, including persistent organic pollutants (POPs) such as PCBs [61] and pharmaceutical contaminants. For example, the anti-inflammatory drug diclofenac, which has been found in domestic livestock carcasses, led to population declines of >95% in Indian vultures (*Gyps* spp.) [62,63]. Besides such toxic effects, chemical pollutants may also have sub-lethal effects on key traits, leading to reduced population sizes and the subsequent opportunity to become invasive. Prior meta-analyses in mammals [56], as well as reptiles and amphibians [17], have demonstrated that species with larger and more frequent litters/clutches have increased introduction success. Importantly, a wide variety of pollutants have been shown to influence reproduction [34,64]. For example, the concentration of metal pollutants in the feathers of great tits (*Parus major*) and blue tits (*Cyanistes caeruleus*) is negatively correlated with reproductive success in the wild [65], while exposure to the insecticide imidacloprid reduced reproduction in female orchard mason bees (*Osmia lignaria*) [66]. Similar results have been found across a variety of taxa, including invertebrates [67,68], fish [69], birds [70] and mammals [71]. By contrast, exposure to other pollutants has been shown to increase traits associated with reproductive output, including total sperm count [72] and fecundity [44]. Taken together, these findings highlight the potential for chemical pollutants to influence local population size and, in turn, the potential to produce invaders, but the specifics depend on the pollutant and species.

Besides demographic considerations such as population density, the traits of individual animals have been shown to play a crucial role in species uptake, transport and introduction [48,73]. For example, individuals that are more active, risk-taking and/or exploratory may be more likely to enter novel transport vectors during accidental [73] or intentional introductions [74]. Importantly, much previous research has shown that these behavioural traits are vulnerable to numerous chemical pollutants [35,36,75]. Similarly, traits that have been proposed as important for surviving suboptimal transport conditions may also be influenced by pollutant exposure. For instance, animals may be exposed to rapid changes in temperature during transport, with invasive species often predicted to be thermal generalists [76]. A variety of pollutants, including pesticides [77] and polycyclic aromatic hydrocarbons (PAHs) found in coal and gasoline [78], can alter thermal tolerance, potentially through changes in metabolic potential [79]. While many pollutants decrease upper thermal maxima, in some cases pollutant exposure may actually increase thermal tolerance [22], potentially making it more likely for exposed individuals to survive changing temperatures during transport.

Interestingly, many of the traits that increase the likelihood of an individual to enter a transport vector may be contrary to those that increase survival and decrease detection during transit [80]. For instance, locomotor activity can be positively associated with increased uptake—as seen in prior research documenting positive selection on genes associated with activity during the uptake stage of the invasive yellow-crowned bishop (*Euplectes afer*) [74]—but is negatively correlated with success during transit [73]. Thus, due to the complex nature of selection during the initial stages of invasion and the varied effects of chemical pollutants on the traits of individuals [35,75], predicting how the interactions between pollutants and trait expression influence introduction success remains challenging without considering case-specific factors.

### (b) Establishment

Only a portion of the animals that are introduced beyond their native range will be able to successfully establish and form viable populations [47,48]. The likelihood of successful establishment depends on a variety of factors, which are both intrinsic (e.g. reproductive strategies, competitive ability, tolerance to environmental stressors) and extrinsic (e.g. habitat and climate suitability, availability of resources, presence of predators and/or competitors) to individuals. Chemical pollution, in this regard, can impact animals in numerous ways that have the potential to profoundly affect the success of non-natives during the establishment stage of the invasion process. Surprisingly, however, there is presently a severe lack of research directly investigating potential impacts of chemical pollution on the potential for non-native populations to establish in new environments, despite the widespread implications for our understanding of the impacts of contaminants on the invasion process.

During establishment, chemical pollutants may alter the likelihood of success or survival of a potential invader in a novel environment. As described above, exposure to chemical pollutants has been shown to alter risk-taking behaviour in a wide variety of taxa [80,81]. Given that risk-taking is often associated with the exploitation of resources, including food and/or shelter [48,82], pollutant-induced changes to risk-taking behaviour may influence the likelihood of non-native species acquiring food and avoiding predators in novel environments. Further, chemical pollutants can also exert strong effects on social behaviours [83]. As increased sociality may assist animals during establishment by reducing Allee effects (i.e. the loss of fitness associated with low population densities) [84], pollutant-induced changes in social behaviour may mediate the ability of non-native species to found self-sustaining populations (but see [83,85,86]).

In addition to surviving beyond their native range, invasive species must successfully reproduce in order to establish viable populations. As outlined above, chemical pollution can influence reproductive success through numerous pathways, including biasing sex ratios, altering gonad development, reducing sperm and egg quality and quantity, and decreasing fertilization success [34]. Contaminants can also influence behavioural processes involved in reproduction by, for instance, modifying mating vigour, changing mating preferences, altering reproductive behaviours and affecting parental care [34,87].

### (c) Spread

Spread is the last stage of successful biological invasion, requiring the invaders to establish a self-sustaining population with individuals that move beyond their initial introduction site [47]. Dispersal tendency and ability are crucial for successful species spread [88], and have been linked to a number of phenotypic traits. This includes, but is not limited to, metabolism [89], body size and growth [90], activity/exploration [19,91], boldness [89], sociability [92] and/or aggression [93].

Growth and relative size seem to be particularly important traits for dispersal tendency [90,94] and organisms often display size- and/or life-stage-dependent sensitivity to particular pollutants [95]. Individuals that are larger or proportioned differently may be more capable of dispersal than smaller individuals. For example, the invasive cane toad (*Rhinella marina*) is a classic case study for dispersal-related traits, with toads at the leading edge of the invasive range exhibiting significantly higher dispersal rates, and a suite of associated changes in morphological (e.g. increased relative limb length and shifts in skeletal structure) and behavioural (e.g. movement linearity) traits compared with those in the central or more-established sites [15,96]. While it is not presently known whether chemical pollutants could influence these particular traits in *R. marina*, there are a number of hypothesized mechanisms through which pollutants might impact growth and development in ways that may influence dispersal. A suite of chemical pollutants have been found to suppress or inhibit growth in exposed individuals [97]. These patterns could result from pollutant-induced feeding suppression, lethargy or reduced foraging success [98]. Additionally, the detoxification of chemical pollutants (e.g. via metabolism or excretion) may impose metabolic or other physiological costs on organisms [43,99] which may, in turn, reduce the energy available for somatic growth.

Differences in body size and growth rate often correlate with dispersal propensity [94], potentially because of smaller individuals being forced out of established habitats by larger, territorial conspecifics. Alternatively, smaller individuals may be less capable of dispersal. In this regard, selective dispersal of individuals of particular size classes may influence and be influenced by mitigation methods, which may or may not effectively impact spread. For example, angling as a strategy to control a non-native population may selectively remove larger individuals from populations or lead to behavioural shifts that reduce individual growth rates [100], resulting in a persistent population of individuals that are smaller in size. Because size and life stage can influence susceptibility to pollutants [95,101], demographically altered populations may differ in their responsiveness to chemical pollutants, and their potential for spread.

A number of other processes may lead to pollutant-moderated dispersal. Indeed, previous field-based research has shown that pharmaceutical [102] and pesticide [103] pollutants can directly affect dispersal and migratory behaviour in Atlantic salmon (*Salmo salar*) and white-crowned sparrows (*Zonotrichia leucophrys*), respectively. Further, the presence of pollutants may encourage movement by organisms away from contaminated habitats through active or passive processes [104–106]. This active or passive avoidance of contaminated habitats may facilitate or inhibit the spread of invasive populations. In this case, movement may precede actual exposure, or sufficient exposure to impact organisms physiologically. For example, aquatic invertebrates may move from the substrate into the water column and ‘drift’ downstream in riparian habitats after pesticides enter the environment [106], which for a non-native species could accelerate range expansion. This may be particularly important in the context of species invasions as organisms are often initially introduced and become established in areas of high human activity where they are likely to encounter chemical pollutants [58]. Alternatively, fish may avoid contaminated environments [104,105], suggesting that contaminated environments could act as barriers that limit range expansion.

## 3. New research frontiers in chemical pollution and invasion biology

Both the influence of chemical pollutants on organisms and how phenotypic trait variation contributes to success through the invasion process are complex. Despite the potential interactive effects between these environmental stressors, very little work has explored the impacts of chemical pollution on animal invasions. Below, we highlight several novel research frontiers and important factors to consider in order to better understand these interactions. These novel directions for future work were selected based on our evaluation of research in chemical pollution and invasion biology, as well as the sparse existing studies that span between these fields. We do not propose this as an exhaustive list of promising future research directions but we do believe that each is likely to influence the cumulative impacts of chemical pollution and biological invasions on wildlife populations.

### (a) Environmentally realistic exposure scenarios

Ecotoxicological assays investigating the impacts of chemical contaminants on organisms have conventionally been performed in standardized laboratory settings with stable light and temperature conditions, often with a single species being exposed over relatively short time periods to individual chemicals of interest [35]. This approach is pragmatic and beneficial in terms of generating reliable and comparable data, although it fails to capture much of the complexity inherent in real-world chemical exposures. For instance, it is the rule rather than the exception that wildlife inhabiting contaminated areas are exposed to complex ‘chemical cocktails’ whose composition varies over space and time, rather than to individual contaminants [107–109]. Further, various contaminants are highly persistent in the environment (e.g. dichlorodiphenyltrichloroethane (DDT), dioxins, PFAS), meaning that organisms can be exposed over protracted time periods [110], potentially across multiple life stages [95,111]. In addition, the detrimental impacts of chemical contamination can interact with other concurrent stressors such as climate change, habitat fragmentation and reduced resource availability [77]. Understanding the impact of environmentally realistic exposures is an understudied priority in ecotoxicology generally, and is virtually unstudied in the context of its impact on the invasion process; yet this approach is critical to understanding (and ultimately predicting) how real biological invasions will be influenced by chemical pollution. Taken together, these characteristics of environmentally realistic exposures can further complicate the task of predicting the impacts of chemical pollution throughout the invasion process. As one example, given that (mixtures of) chemical contaminants in real-world ecosystems characteristically vary in concentrations and composition over time and space, contamination could theoretically occur within and across multiple stages of the invasion process, potentially including any combination of these stages (see electronic supplementary material, figure S1 for an illustrative example). Despite the clear potential implications of contaminant exposure within and across stages of the invasion process on invasion success, surprisingly little research has been done to disentangle this issue, which is especially significant given the ubiquitous presence of a wide variety of chemical contaminants in ecosystems globally [112].

However, assessing every potential chemical detected in the environment and their combinations for potential phenotypic effects would be impractical due to high costs, inefficiency and ethical concerns. To this end, environmental sampling for contaminants at locations known to be hotspots for invasions (e.g. areas of high biodiversity, economic and transport hubs [113–115]) provides a more targeted approach in identifying those chemicals that may be most likely to influence invasion success. When paired with *in silico* tools, such as data-driven computational models, machine learning algorithms and molecular networking [109,116,117], which can use structural information to predict the effects of environmentally detected chemicals (e.g. endocrine disruption [118]), these more targeted approaches may allow for the identification of contaminants (and their combinations) at invasion hotspots that are likely to influence those traits known to be important during biological invasions—a critical task in advancing our understanding of how chemical pollutants can alter invasion success across ecologically realistic scenarios.

### (b) Direct and indirect effects of pollutants and interactions between native and non-native species

In addition to effects within species, chemical pollution can also affect interactions with heterospecifics. While heterospecific interactions have been an important focus in the study of invasion ecology [119,120], how these interactions are influenced by exposure to chemical pollution remains largely unexplored. Despite a lack of explicit research on potential interactive effects of chemical pollutants and heterospecific interactions between native and introduced species, there are several ways such interactive effects could emerge. Perhaps the most important heterospecific interaction to consider in this framework is competition because, when facing polluted environments, some non-native species may possess a competitive advantage over natives. For example, copper exposure resulted in reduced native species richness, but did not impact invasive species richness on fouling plates deployed in a marine environment [29]. This can occur if, for example, non-native species are more tolerant to pollutants than natives. Indeed, certain invasive species may have higher immune function and experience a release from parasitism [121,122], the consequences of which could be amplified in polluted environments [123]. By contrast, native species—which have a longer history of exposure to the chemically contaminated environment they inhabit—may be locally adapted to these conditions [124], leaving them more resistant compared with non-adapted non-natives and giving them the competitive edge. This is made more complex by the fact that limited energy budgets typically imply fitness costs to evolved pollutant tolerance. For example, evolved tolerance to the insecticide carbaryl was associated with an increased susceptibility to parasites in *Daphnia magna* [125], which, in a multi-stressor environment, may tip the scale in favour of non-adapted individuals. More generally, competitive outcomes (between natives and non-natives) are likely to be affected by exposure to chemical pollutants, and may depend on whether the stronger competitor is more or less sensitive to contamination than the weaker competitor, as well as the costs of such tolerance [126].

Furthermore, chemical pollution can indirectly affect the chances of non-natives establishing by altering the environment in ways that differentially impact various species in the community. For example, chemical pollution can impact the availability of food for native and non-native species alike [127], and generalist non-natives may be better able to succeed in such environments. Physiological and morphological traits could also be indirectly affected following changes in resource availability as a result of chemical pollution in ways that benefit non-native animals over their native counterparts [128]. Predation is another interspecific interaction that is both highly relevant to the success of biological invasions and known to be altered by various pollutants. Non-native predators establishing in a polluted environment may encounter prey that are affected by pollution, which could indirectly affect their predation success, positively or negatively, and thus the establishment of non-native predators [76]. On the other hand, non-native prey species could benefit from polluted environments and thrive to become invasive pests, overcoming the predation pressure that native predators may pose.

Clearly, both direct and indirect effects of contaminants on any of these processes in invading and/or native species during establishment stand to influence the success or failure of the invasion process. Such effects can cascade across trophic levels, leading to unforeseen and often unpredictable outcomes for the functioning of ecosystems and their services. Consideration of these interactive processes will facilitate a more realistic understanding of the influence of chemical pollution on the success of biological invasions and potentially more generalizable predictions regarding how these interactive processes emerge in nature.

### (c) The invasion process as a selective filter

As outlined above, biological invasions are a multi-stage process (uptake, transport, introduction, establishment and spread [47]). At each of these stages, potential invaders can either succeed or fail, resulting in the invasion process acting as a sequential selective filter on species traits that are associated with invasion success (‘selective filter hypothesis’ [48,81]). This selective filtering is, thus, thought to result in a non-random subsample of invasive individuals with particular trait combinations. Where these traits have a heritable basis, this likely decreases both phenotypic and genetic variation in invasive populations [81,129].

Importantly, exposure to chemical pollutants may disrupt selective filtering during invasions in ways that change the population of successful invaders. For example, numerous chemical pollutants have been shown to increase activity and exploratory behaviour in a variety of aquatic species [36,57]. As discussed previously, this could result in a greater number of individuals being likely to enter transport vectors within their native range, resulting in a greater number of individuals being introduced than otherwise would have in the absence of the pollutants. Crucially, this exposure-induced increase in exploratory behaviour may occur regardless of the underlying genotype, resulting in reduced genotype–phenotype associations. In such cases, pollutant exposure may allow a greater number of genotypes ultimately being introduced into areas beyond their native range, leading to higher genetic diversity in the invasive population than could have occurred without the pollutant. Thus, it may not just be a larger number of invaders, but the invaders may be more phenotypically diverse when they enter a new environment and are no longer under the influence of the same chemical pollutants. This phenotypic diversity could increase the ability of a species to respond to new challenges in the environment, making invasions more likely to be successful. While this is just one simplified hypothetical example, it is clear that investigating how chemical pollution may alter the selective filtering of phenotypes during invasions is a key topic of future research.

### (d) The role of phenotypic plasticity and behavioural syndromes

Phenotypic plasticity—the expression of multiple phenotypes from one genome—allows organisms to respond rapidly to environmental change. This, in turn, can either facilitate or hinder evolutionary (genetic) adaptation, which determines the persistence of populations in the long term [129]. Interestingly, at the population scale, traits that are highly plastic in the face of changing environments are often also characterized by greater than expected additive genetic variation [130] and thus shifts in phenotypic expression by organisms exposed to chemical pollution could be the result of plasticity or selection for (or against) existing genetic variation. Evidence suggests that successful invaders tend to be more flexible in a variety of (often correlated) phenotypic traits, including behaviour, morphology, life history and physiology [85,131]. Such flexibility is likely to give invaders an advantage when competing with native populations or species if such flexibility translates into fitness benefits [89]. Whether and how phenotypic plasticity is itself shaped by exposure to chemical pollutants has received relatively little research attention (but see [132–134]). Still, the impacts of chemical pollutants on intra- and inter-individual phenotypic variation are potentially far more widespread than is currently appreciated. For example, exposure to the psychoactive pharmaceutical pollutant fluoxetine can alter the plasticity of behaviour [132,133], metabolic rate and even their individual-level correlations [134] in guppies (*Poecilia reticulata*)—highlighting the potential for pollutant-induced changes in plasticity to affect invasion success.

It is important to note that phenotypic plasticity may also hinder (genetic) adaptation to novel environments, limiting the invasive potential of populations or species. For example, when plastic responses fail to keep pace with rapid and/or unpredictable environmental change, this may lead to phenotype–environment mismatches that are maladaptive and that, in turn, favour invaders and/or native populations that are less plastic [129]. The fact that phenotypes associated with range expansion, including phenotypic plasticity, play a key role in facilitating species transport, introduction, establishment and spread, suggests that the process of invasion could also act as a selective filter on such traits. For example, the selection of more-exploratory individuals during the introduction of the invasive delicate skink (*Lampropholis delicata*) was associated with an increase in behavioural plasticity in invasive skinks relative to their native-range counterparts [80]. Yet it is important to note that different ecological conditions encountered by invasive species in their new environments can alter the association between phenotypic traits at the individual level altogether (i.e. disrupt behavioural and pace-of-life syndromes [19,135,136]). For example, while activity level and foraging rate are correlated in domestic Siamese fighting fish (*Betta splendens*), this relationship was absent in invasive populations [19]. Even though both the biological invasion process and chemical pollution can profoundly affect the expression and selection of phenotypes, how these two phenomena may jointly shape the outcomes of interactions at range-expansion fronts is still elusive.

## 4. Conclusions

The introduction of non-native species and exposure to chemical pollutants represent two of the most concerning threats facing wildlife populations today. While substantial work has investigated the potential for environmental changes such as temperature and carbon dioxide levels to influence the spread of non-native species [32,33], the potential influence of chemical pollution on invasion success remains largely unexplored. While chemical pollutants are themselves diverse and the potential impacts that they may have on wildlife have not yet been fully established, we highlight several potential mechanisms through which interactive effects could emerge at different stages of the invasion process. Importantly, these impacts are not homogeneous; for example, pollutant-mediated changes that might facilitate the success of a non-native species entering novel environments may be detrimental at other stages.

In this review, we explore mechanisms through which pollutants might influence the success of non-native species. Well-documented examples of chemical pollutants influencing development, metabolism, reproductive physiology and behaviour provide a foundational framework of how chemical pollutants might moderate the introduction, establishment and spread of non-native species. While many questions remain unanswered regarding both the impacts of chemical pollutants on wildlife and the contributing factors and processes that underlie the successful spread of non-native species, in this review we highlight many promising examples of systems in which potential testable predictions can be explored. Building on this framework and that of other researchers exploring the interactive effects of environmental change on organisms and ecosystems, future work on emergent pollutants of interest, chemical mixtures (which most wildlife experience) and the influence of other environmental stressors can be integrated. It is important to note that we largely focus on how chemical pollutants can influence the success of unintentional introductions and invasions. However, deliberate introductions are also associated with distinct challenges—particularly during the early stages of the invasion (e.g. trappability, survival in captivity [137])—that may be influenced by an organism’s exposure to chemical pollutants. Thus, identifying how chemical pollutants can mediate the success of deliberate introductions will be a key avenue for future work. While challenging, understanding how these factors collectively influence wildlife is of critical importance for effectively planning and carrying out conservation efforts for animal populations.

## Data Availability

Supplementary material available online [138].
